# Making sense of cilia: The role of intraflagellar transport

**DOI:** 10.1371/journal.pbio.3002924

**Published:** 2024-11-27

**Authors:** Renny Ng, Chih-Ying Su

**Affiliations:** Department of Neurobiology, University of California San Diego, La Jolla, California, United States of America

## Abstract

Intraflagellar transport (IFT) is essential for both ciliary structure and function. A new study in *PLOS Biology* reveals how IFT-mediated trafficking and ciliary morphology differentially influence chemosensory responses between neuronal types and among co-expressed receptors.

The interplay between neuronal form and function presents an intriguing puzzle for neurobiologists. How does the morphology of a sensory neuron affect its ability to detect stimuli? This question is especially compelling in ciliated sensory neurons, which exhibit diverse morphological features in a modality-specific manner [1]. Since cilia lack the machinery to synthesize proteins, intraflagellar transport (IFT) is responsible for transporting structural and signaling molecules across the ciliary gate and trafficking them along the cilia [2]. Thus, permanent mutation of key IFT machinery not only prevents signaling molecules from being trafficked to the cilia, but also impairs trafficking of structural molecules required for normal cilia structure. As such, a complete understanding of how IFT and ciliary morphology individually shape sensory responses remained elusive.

In the current issue of *PLOS Biology*, Philbrook and colleagues explored how chemosensory responses are differentially affected when ciliary trafficking and sensory ending morphology are selectively manipulated [3]. Using *Caenorhabditis elegans* as a model, the researchers engineered temperature-sensitive (ts) mutations in IFT motor proteins to acutely disrupt IFT without altering ciliary morphology and vice versa. This innovative genetic approach disentangled the effects of continuous trafficking from ciliary structure to reveal their respective contributions in shaping the responses of 2 ciliated sensory neurons—the ASH nociceptive neurons and AWA olfactory neurons [4].

ASH neurons extend rod-like cylindrical cilia that detect noxious chemicals such as glycerol (Fig 1A, left panel). In *kap-1; osm-3(ts)* kinesin mutants, raising the temperature to 27 °C for 1 h acutely truncated cilia without disrupting continuous IFT and also diminished responses to glycerol (Fig 1A, middle panel). In contrast, placing *che-3(ts)* dynein mutants in a restrictive temperature for hours disrupted IFT without shortening cilia. Despite impaired IFT, these neurons exhibited wild type-like glycerol responses (Fig 1A, right panel) that likely arise from receptors and signaling components already trafficked to ASH cilia prior to temperature-induced IFT disruption, which in turn suggests a limited turnover for these molecules in the cilia. Overall, normal ASH glycerol responses require a minimum ciliary length, rather than continuous IFT.

**Fig 1:**
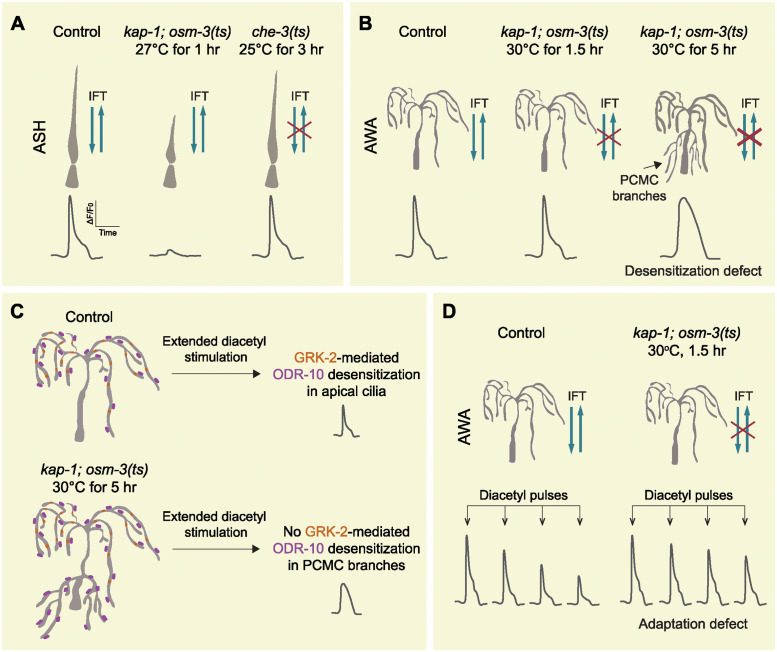
Ciliary structure and IFT differentially impact the responses of ASH nociceptive and AWA olfactory neurons. (**A**) In ASH neurons, reduced ciliary length diminishes responses to ligands, while disrupted IFT does not. (**B**) In AWA neurons, prolonged IFT disruption leads to formation of PCMC branches. This coincides with a desensitization phenotype to diacetyl, an odorant that activates ODR-10 receptor. (**C**) Mechanistic model: Extended odor stimulation triggers GRK-2-mediated desensitization of ODR-10 in AWA cilia. In the absence of IFT, ODR-10—but not GRK-2—is localized to PCMC branches, preventing receptor desensitization in these ectopic branches. (**D**) Acute IFT disruption impairs AWA adaptation to repeated diacetyl stimulation.

Is this also the case with AWA olfactory neurons, which detect various volatile odorants through multiple receptors expressed in their highly branched sensory cilia (Fig 1B, left panel)? Interestingly, in the same *kap-1; osm-3(ts)* mutants, shifting to a restrictive temperature resulted in a strikingly different phenotype in AWA neurons. Specifically, raising the temperature to 30 °C for 1.5 h abolished IFT without altering ciliary morphology, and this manipulation likewise did not affect responses to diacetyl, an odorant that activates the ODR-10 receptor (Fig 1B, middle panel). However, lengthier disruption of IFT (30 °C for 5 h) led to formation of ectopic dendritic branches from the periciliary membrane compartment (PCMC), which coincided with a desensitization phenotype whereby responses decayed more slowly than in controls (Fig 1B, right panel). These findings suggest that abolishing IFT for hours does not reduce AWA diacetyl responses, while desensitization is impaired when PCMC branches are formed.

Mechanistically, these phenotypes are explained by the differential regulation of ODR-10 desensitization in PCMC branches and AWA cilia. Desensitization in wild-type neurons arises through phosphorylation of ODR-10 by the GRK-2 GPCR kinase, which inactivates the receptor to drive rapid response decay. Both ODR-10 and GRK-2 are co-localized in AWA cilia, even after 5 h of IFT disruption in *kap-1; osm-3(ts)* mutants. However, when IFT is impaired, only ODR-10 but not GRK-2 is localized to the newly formed PCMC branches. As a result, while mislocalized ODR-10 in these ectopic branches can respond to diacetyl, these receptors cannot be properly phosphorylated and then inactivated during extended odor stimulation, leading to the desensitization phenotype (Fig 1C). These findings highlight how odorant response kinetics are shaped by the spatial organization of signaling molecules.

Adding to the complexity, SRX-64, another AWA olfactory receptor responding to pyrazine, is not mislocalized to PCMC branches as robustly as ODR-10 in the absence of IFT. Interestingly, continuous IFT is required for ODR-10 adaptation to repeated diacetyl stimulation (Fig 1D), but not for SRX-64 adaptation to pyrazine pulses. These findings reveal a surprisingly complex and heterogenous role of IFT in trafficking molecules and regulating adaptation, even among receptors co-expressed in the same olfactory neurons.

By revealing the distinct roles of IFT and ciliary morphology in *C*. *elegans* chemosensory responses, Philbrook and colleagues have advanced our understanding of the intricate interplay between form and function in sensory neurons. This in-depth genetic study paves the way for future research to explore the molecular mechanisms by which IFT and ciliary architecture regulate sensory responses in a receptor-, ligand-, and neuron-specific manner. It will be interesting to investigate whether similar functional heterogeneity exists in other sensory neurons across different animal species.

This study carries broad implications, as cilia are crucial for olfactory receptor neurons, and defects can lead to ciliopathies that compromise the sense of smell [5]. For instance, COVID-19-related anosmia likely arises from the retraction of olfactory cilia following the death of SARS-CoV-2 infected support cells [6]. Does IFT disruption underlie this change in ciliary morphology in COVID-19 patients? It will also be interesting to determine whether IFT’s heterogeneous effects in *C*. *elegans* olfactory responses are mirrored in different populations of human olfactory receptor neurons.
